# Molecular and Functional Alterations of P-Glycoprotein in a Genetic Model of Epilepsy: Insights from the Wistar Audiogenic Rat

**DOI:** 10.3390/ijms27083544

**Published:** 2026-04-16

**Authors:** Rodrigo V. Placido, Rafaela F. Rodrigues, Lellis H. Costa, Taila Kawano, Milton K. Aquino, Gabriella B. Martinez, Mariana T. R. Hummel, Maria Eduarda T. de Lima, Rui M. P. da Silva, Norberto Garcia-Cairasco, Silvia G. Ruginsk, Marília G. A. G. Pereira, Vanessa B. Boralli

**Affiliations:** 1 Pharmaceutical Sciences Faculty, Federal University of Alfenas, Alfenas 37130-001, MG, Brazil; rafaelafigueiredor@gmail.com (R.F.R.); lellis.costa@hotmail.com (L.H.C.); tailakawano@gmail.com (T.K.); miljunior1@gmail.com (M.K.A.J.); bechler.gabriella@gmail.com (G.B.M.); marithaliahummel@gmail.com (M.T.R.H.); mariaeduardatl05@gmail.com (M.E.T.d.L.); 2 Neurophysiology and Experimental Neuroethology Laboratory, Department of Physiology, University of São Paulo, Ribeirão Preto 05508-220, SP, Brazil; ruimiltonpsj@hotmail.com (R.M.P.d.S.J.); ngcairas@usp.br (N.G.-C.); 3 Institute of Biomedical Sciences, Federal University of Alfenas, Alfenas 37130-001, MG, Brazil; silvia.leitao@unifal-mg.edu.br (S.G.R.); marilia.pereira@unifal-mg.edu.br (M.G.A.G.P.)

**Keywords:** epilepsy, drug resistant epilepsy, pharmacokinetics, ATP binding cassette transporter, subfamily B, member 1, drug resistance

## Abstract

Drug resistance remains a major challenge in epilepsy, and overexpression of ATP-binding cassette transporters, particularly P-glycoprotein (P-gp), at the blood–brain barrier (BBB) has been consistently implicated in limiting central nervous system drug exposure. Genetic experimental models suitable for investigating molecular regulation and functional alterations of P-gp in epilepsy remain scarce. This study evaluated P-gp expression and functional alterations in the Wistar Audiogenic Rat (WAR), a genetic model of epilepsy exhibiting phenotypic heterogeneity. WAR animals were classified into refractory epilepsy (WAR-RE) or temporal lobe epilepsy (WAR-TLE) phenotypes and compared with non-epileptic Wistar controls. Fexofenadine, a well-established in vivo P-gp probe substrate, was administered orally, and plasma pharmacokinetic parameters were determined. P-gp expression at the BBB was assessed by immunohistochemistry in hippocampal regions. WAR-RE animals exhibited significantly increased systemic exposure to fexofenadine, characterized by higher area under the curve and prolonged half-life, alongside reduced apparent clearance, compared with control animals (*p* < 0.05). In contrast, WAR-TLE animals showed greater interindividual variability without statistically significant differences. Immunohistochemical analysis revealed increased P-gp expression in hippocampal microvessels in both WAR phenotypes. These findings demonstrate that the WAR model displays molecular upregulation of P-gp at the BBB, accompanied by functional alterations in the disposition of a prototypical P-gp substrate. Although direct brain drug concentrations were not assessed, the integration of systemic pharmacokinetics with transporter expression supports the use of WAR as a genetic proof-of-concept model for studying P-gp regulation and transporter-mediated drug disposition in epilepsy. This model provides a valuable molecular framework for future investigations addressing transporter modulation and mechanisms underlying pharmacoresistance.

## 1. Introduction

Epilepsy is one of the most common neurological disorders worldwide, affecting approximately 50 million people and an estimated 1 to 3% of the global population [1]. It is characterized by recurrent and spontaneous seizures caused by abnormally excessive or synchronous electrical discharges in brain neurons, resulting in brief episodes of involuntary movement, known as seizures [2,3]. These seizures may affect a specific region of one cerebral hemisphere (partial epilepsy) or both hemispheres (generalized epilepsy).

Drug treatment is essential for improving patients’ quality of life [4]; however, approximately 30% of individuals with epilepsy, particularly those with temporal lobe epilepsy (TLE), do not respond adequately to pharmacological therapy and are therefore classified as having drug-resistant epilepsy [1,5].

P-glycoprotein (P-gp) is an ATP-dependent efflux transporter first described in the 1970s [6] that is found in the blood–brain barrier (BBB), intestine, kidneys, and other organs [7,8]. It plays a critical role in limiting drug absorption and distribution, and its expression can be induced by certain diseases, such as cancer, or by specific drugs [9,10].

In the context of epilepsy, pharmacoresistance remains a complex and clinically significant issue, requiring careful evaluation by a multidisciplinary healthcare team. Among the proposed mechanisms, the overexpression of efflux transporters, particularly P-gp at the BBB, is one of the most extensively studied hypotheses [11]. This protein can hinder therapeutic efficacy by preventing antiepileptic drugs from reaching effective concentrations in the central nervous system. Notably, many antiepileptic drugs are both substrates and inducers of P-gp [12], reinforcing its potential contribution to treatment failure. These pharmacoresistance hypotheses and supporting preclinical evidence have been critically reviewed by Servilha-Menezes and Garcia-Cairasco [13], highlighting the importance of investigating P-gp function and regulation in experimental epilepsy models.

The genetic model we use is called Wistar Audiogenic Rat (WAR) [14]. Acute audiogenic seizures are evoked by high-intensity acoustic stimulation (120 dB), and the specific phenotype is wild running behaviors (running, jumping, and atonic falling) followed by tonic–clonic seizures, which are usually dependent on the activity of brainstem structures [15]. Susceptibility appears around the sixth week of life, and this trait is heritable and stable across generations. Because it is a genetic model, their response is closer to that of humans [14,16]. Importantly, the WAR model exhibits marked phenotypic heterogeneity following audiogenic kindling, allowing the distinction between animals with limbic recruitment and those with limited seizure generalization. Although the WAR strain is genetically selected for susceptibility to audiogenic seizures, this heterogeneity emerges during audiogenic kindling due to differential recruitment of neural networks and variability in epileptogenic plasticity. Thus, genetic uniformity at baseline does not preclude divergence in seizure progression or limbic engagement following repeated stimulation. This feature makes the WAR strain particularly suitable for investigating molecular mechanisms associated with differential drug disposition and transporter regulation under comparable genetic conditions.

In fact, the WARs have been genetically selected since the 80s at the University of São Paulo, Department of Physiology, Neurophysiology and Experimental Neuroethology Laboratory, Ribeirão Preto, São Paulo, Brazil, by endogamic mating (sister × brother) over more than 65 generations. Subsequently, they were rederived to be exported internationally as WAR*spf*. The latter process was made at the CEMIB-University of Campinas, Brazil, and they are available internationally since 2022, through a Material Transfer Agreement (MTA) between the University of São Paulo and the Rat Resource and Research Center (RRRC) at the University of Missouri (USA) as RRRC# 697: W/LnneRrrc.

Interestingly, when WARs are exposed to repeated audiogenic seizures, the so-called audiogenic kindling [17], there is a clear decrease in the presence of brainstem seizures, coincident with the appearance of typical forebrain–limbic seizures, similar to those evoked by electrical stimulation of amygdala or hippocampus [18,19,20].

Despite the extensive use of the Wistar Audiogenic Rat (WAR) model in studies of epileptogenesis and seizure susceptibility, its applicability for investigating pharmacoresistance mechanisms, particularly those involving efflux transporters at the blood–brain barrier, remains largely unexplored. To the best of our knowledge, the hypothesis that overexpression of efflux transporters, such as P-gp, contributes to pharmacoresistance has not yet been directly tested in this genetic model (WAR), nor have pharmacokinetic investigations been conducted. Therefore, the present study aimed to investigate molecular and functional alterations associated with P-glycoprotein regulation in the WAR model, using a validated pharmacokinetic probe substrate to explore transporter-related mechanisms in a genetic model of epilepsy.

## 2. Results

### 2.1. Pharmacokinetics of Fexofenadine

**Figure 1:**
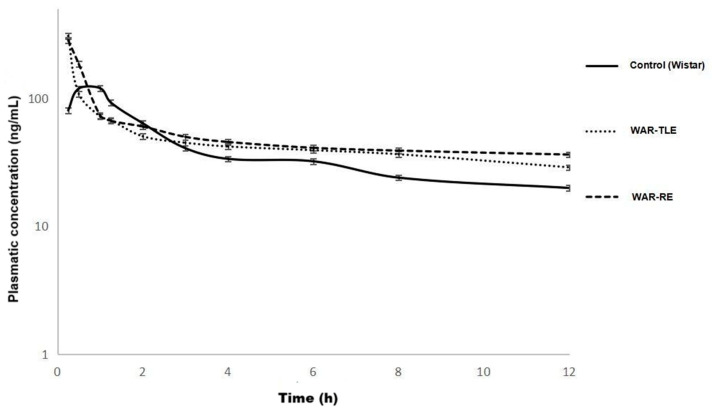
Plasma concentration–time profiles of fexofenadine (10 mg/kg, oral single dose) in control (Wistar), WAR-TLE, and WAR-RE animals. Data illustrate systemic pharmacokinetic differences among experimental groups.

The comparative analysis between groups revealed significant differences in the parameters AUC (577.9 vs. 1200.8 ng/mL·h, *p* < 0.05), t_1_/_2_ (4.2 vs. 11 h, *p* < 0.05), and clearance (17,396 vs. 8330 L/Kg/h, *p* < 0.05) in WAR-RE, compared to the control group (Table 1 and Figure 1). Clearance values are reported as apparent total clearance (Cl_T/f_), as fexofenadine was administered orally, and therefore reflect both systemic elimination and differences in oral bioavailability.

The WAR-TLE group showed a tendency toward increased bioavailability and a longer plasma half-life compared to the control group, but the differences were not significant. In addition, there was no difference in pharmacokinetic parameters when compared to the resistant epilepsy group (Table 1 and Figure 1).

Notably, the concentration–time profile of the WAR-RE group shows a visibly prolonged elimination phase compared to the control group, consistent with the increased half-life and reduced apparent clearance observed.

These differences reflect alterations in the systemic disposition of a prototypical P-gp substrate and do not directly indicate changes in central nervous system drug exposure.

### 2.2. Immunohistochemistry

**Figure 2:**
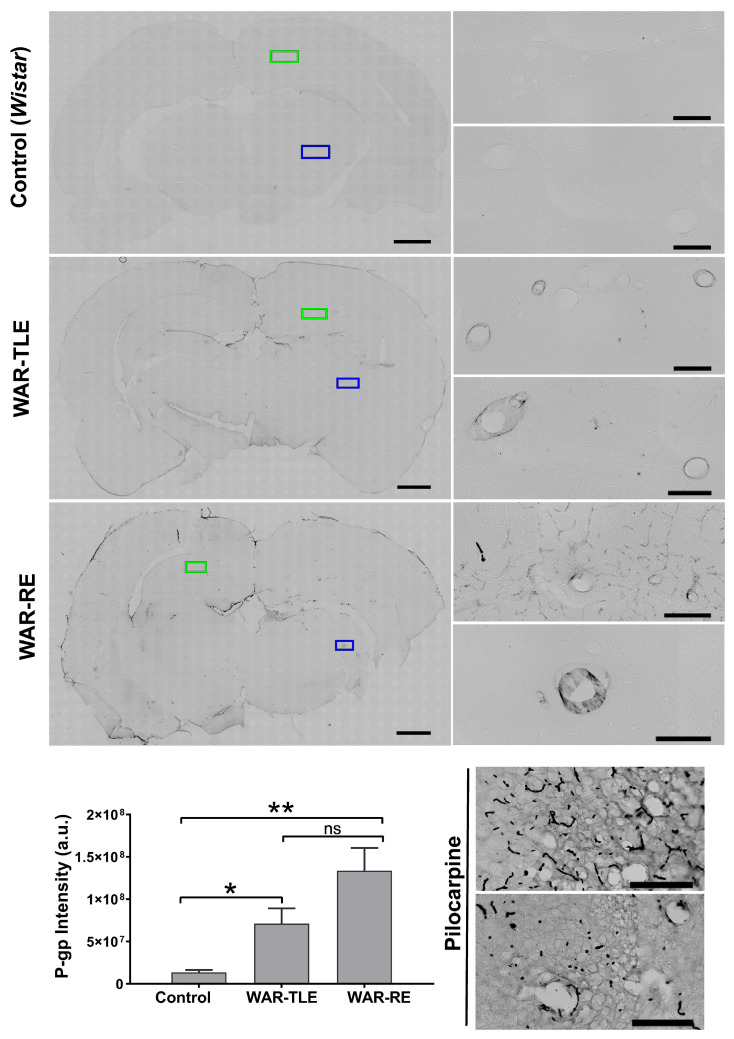
Immunohistochemical analysis of P-glycoprotein expression in hippocampal microvessels from control (Wistar), positive control (pilocarpine-induced epilepsy), WAR-TLE, and WAR-RE animals. Representative images are shown. P-gp immunoreactivity was semi-quantitatively assessed by densitometric analysis. Green and blue regions of interest (ROIs) indicate representative hippocampal microvascular areas used for densitometric quantification of P-gp expression. Statistical difference found in WAR groups with TLE and RE in relation to the control group. * *p* < 0.05, ** <0.01, ns = not significant, One Way ANOVA test, Black Line: Scale bar: 50 µm.

Immunohistochemistry confirmed increased expression of P-gp in the WAR-TLE and WAR-RE groups, comparable to the positive control (pilocarpine-induced epilepsy), mainly in small and medium-sized blood vessels (Figure 2). P-gp expression was quantified by densitometric analysis of immunoreactive areas, allowing semi-quantitative comparison of staining intensity among experimental groups.

## 3. Discussion

The present findings indicate an association between increased P-gp expression at the blood–brain barrier and altered systemic pharmacokinetics of a validated P-gp substrate in the WAR model. This integrative approach strengthens the molecular interpretation of transporter regulation in a genetic epilepsy model. The pharmacokinetic parameters observed in the control group (oral dose of 10 mg/kg) are in line with the values reported by Phatak et al. (t_1/2_ = 3.12 h, t_max_ = 0.75 h) [21] and Peng et al. (t_1/2_ = 4.96 h, t_max_ = 1.33 h) [22], proving the reliability of the method applied.

Changes in the pharmacokinetic parameters of the WAR-RE group show greater systemic bioavailability and lower elimination of fexofenadine compared to the control group. These results suggest pharmacokinetic changes potentially associated with P-glycoprotein overexpression in the WAR model. These findings should be interpreted in the context of the selected brain region. The hippocampus was chosen due to its central role in epileptogenic remodeling and limbic seizure propagation, particularly in temporal lobe epilepsy, modeled in the context of this study by the audiogenic kindling protocol in WAR-TLE [19,20]. Therefore, this regional focus reflects an emphasis on mechanisms associated with epileptogenesis and pharmacoresistance rather than acute brainstem-mediated seizure propagation.

It is important to recognize that changes in oral AUC and apparent clearance may result from multiple determinants beyond transporter activity, including hepatic metabolism, renal excretion, plasma protein binding, and oral bioavailability. Therefore, the observed pharmacokinetic alterations likely reflect the combined influence of transporter regulation and systemic factors.

In addition to P-glycoprotein, other ATP-binding cassette transporters such as BCRP (ABCG2) and members of the MRP family have been implicated in drug-resistant epilepsy and may interact with P-gp to shape drug disposition at the blood–brain barrier.

Beyond transporter abundance, several molecular mechanisms have been implicated in the regulation of P-glycoprotein expression and activity in epilepsy. Seizure-induced neuroinflammation, activation of nuclear receptors, epigenetic modifications, and microRNA-mediated pathways have been shown to dynamically modulate ABCB1 transcription and transporter function. These regulatory processes may contribute to the phenotypic heterogeneity observed in the WAR model and help explain the differential pharmacokinetic profiles between WAR-RE and WAR-TLE animals.

Although WARs-TLE showed no mean change in AUC compared to controls, the large individual variation suggests that the presence of epilepsy alone is not determinative for pharmacokinetic changes. In contrast, WARs-RE showed significantly higher bioavailability and reduced clearance, suggesting a functional effect of P-gp in these animals.

It is important to emphasize that increased systemic exposure to a P-gp substrate does not necessarily translate into increased brain penetration. In the context of blood–brain barrier overexpression of P-gp, higher plasma AUC values may reflect restricted central nervous system entry, reinforcing the role of efflux transporters in limiting drug availability at the epileptic focus.

Clinically, approximately 30% of patients with epilepsy do not respond to conventional drug therapy, reflecting the complexity of factors involved: genetic, epigenetic, and environmental. The WAR model, despite being genetically homogeneous, reproduces this phenotypic heterogeneity presented by refractory patients [14]. This phenotypic variability was also reflected in the bioavailability of fexofenadine, reinforcing the role of efflux transporters in individualized therapeutic response, simulating the severity of the disease and the difficulty of diagnosis and treatment of patients [1].

The increase in P-gp expression in both WAR groups is consistent with the presence of a functional barrier limiting the penetration of substrates such as fexofenadine into the central nervous system (CNS), resulting in higher plasma AUC without pharmacological effectiveness. The use of this drug as a research tool is noteworthy, but similar behavior can be observed with other centrally acting drugs, contributing to persistent treatment failures.

Importantly, this study represents the first pharmacokinetic and functional evaluation of P-glycoprotein activity in the WAR model. By integrating systemic pharmacokinetics with transporter expression at the blood–brain barrier, the WAR strain emerges not only as a model of epileptogenesis but also as a robust experimental platform for investigating mechanisms of pharmacoresistance.

In a preclinical study using a chemical epilepsy model, the modulating agent fingolimod was shown to reduce P-gp expression, increase brain phenobarbital concentrations, and thus attenuate seizures in drug-resistant animals, suggesting a promising therapeutic avenue [23].

In line with the data found in the present study, preclinical data have shown that P-gp is increased in limbic regions after status epilepticus [24], and its inhibition with tariquidar restored the efficacy of phenytoin in a model of refractory epilepsy [25]. Other studies in genetic and chemical models have reported increased expression of transporters such as P-gp and decreased influx transporters [7,26,27,28]. Also, recent studies have demonstrated the role of microRNAs (miRNAs) targeting P-gp in the diagnosis of intractable epilepsy [29].

In addition, recent reviews advocate a complex systems approach to understanding refractory epilepsy, integrating transporters, inflammation, and phenotypic expressions [13]. There is emerging clinical evidence that overexpression of P-gp significantly contributes to drug resistance in patients with epilepsy. Positron emission tomography (PET) ([^11^C] metoclopramide) studies identified a functional increase in P-gp in the blood–brain barrier of refractory individuals [30]. Clinical and experimental reviews highlight the decreased efficacy of antiepileptic drugs as a consequence of the elevated expression of transporters [31]. Furthermore, pharmacological interventions such as fingolimod have been shown in animal models to reduce P-gp, improve brain drug distribution, and attenuate drug refractoriness [32]. Studies in human tissues have also identified increased levels of P-gp in epileptic regions of patients with refractory epilepsy, strengthening the translational character of the observed mechanism [33].

This study has limitations. Brain drug concentrations were not directly measured, classical antiseizure medications (ASM) were not evaluated, and pharmacological inhibition of P-glycoprotein was not performed. Therefore, the present findings should be interpreted as molecular and functional associations rather than direct evidence of transporter-mediated antiepileptic drug resistance.

Future studies integrating brain-to-plasma drug concentration ratios, pharmacodynamic outcomes, and selective modulation of transporter activity will be essential to further elucidate the role of P-glycoprotein in epilepsy-associated drug resistance. In this context, the WAR model provides a valuable genetic platform for testing pharmacological, molecular, and epigenetic strategies aimed at modulating transporter function before clinical investigation.

## 4. Materials and Methods

### 4.1. Study Design and Animal Groups

Wistar rats (*n* = 6, each group) were obtained from the Central Animal Facility of the Federal University of Alfenas, and animals of the WAR strain (*n* = 6, each group) were obtained from the Department of Physiology, Ribeirão Preto School of Medicine, University of São Paulo. A total of 18 animals were included in the study, distributed into three experimental groups (*n* = 6 per group). The sample size was defined based on previous exploratory pharmacokinetic and transporter expression studies in experimental epilepsy models, in which similar group sizes were sufficient to detect biologically relevant differences. All animals were 7 weeks old and weighed between 200 and 250 g. The animals had free access to standard feed and water and were kept at 22 °C on a 12:12 h light/dark cycle. All experiments were conducted during the light cycle.

Experiments were conducted following the US National Institutes of Health Guidelines for the Care and Use of Laboratory Animals and were approved by the Institutional Animal Care and Use Committee before commencement (CEUA/UNIFAL-MG protocol number 658/2015).

The experimental groups were (1) control (Wistar), (2) WAR-RE (Wistar Audiogenic Rats subjected to kindling that presented up to three mesencephalic seizures), and (3) WAR-TLE (Wistar Audiogenic Rats subjected to kindling with limbic recruitment, presenting at least three limbic seizures during audiogenic kindling) [34]. All groups received fexofenadine (P-gp substrate—10 mg/Kg orally, by gavage), with doses based on previous studies [35,36].

### 4.2. Audiogenic Kindling Model

The audiogenic kindling protocol was performed with acoustic stimulation twice a day [17,18,19,37] at fixed times (between 08:00–09:00 h and 16:00–17:00 h) during 10 consecutive days.

Briefly, an acoustically isolated box was used to expose each animal to a high-intensity sound until a tonic seizure appeared or for a maximum time of 1 min [37]. The sound of a ringing bell (120 dB) was digitized with a high-pass filter (N500 Hz) and reproduced with a personal computer coupled to amplifiers and tweeters on the top of the cage. The severity of tonic–clonic seizures was evaluated using the mesencephalic severity index [34]. Racine’s scale (limbic index) was used to assess the severity of temporal lobe (recruited) epileptic seizures [38]. To include animals in the group WAR-TLE, the rats were required to show at least three limbic seizures (more than level 3 in limbic index) during audiogenic kindling. To include animals in the WAR-RE group, the rats were required to show up to three tonic–clonic seizures (less than level 3 in mesencephalic index) during the audiogenic kindling. Wistar rats (control) do not present any seizures after repeated stimuli.

Thus, WAR-TLE animals represent a phenotype with limbic recruitment and temporal lobe seizure expression, whereas WAR-RE animals represent a phenotype with limited limbic engagement and resistance to seizure generalization despite repeated stimulation.

### 4.3. Plasma Pharmacokinetic Studies

Seven days after the last kindling stimulation, all animals were cannulated in the jugular vein for blood collection and were placed in individual boxes [39]. Fexofenadine (Sanofi, Campinas, Brazil) 10 mg/kg, orally (gavage), single dose) was administered 12 h after the procedure, and 500 μL of blood was collected in heparinized tubes after 0.25, 0.50, 1, 1.25, 2, 3, 4, 6, 8, and 12 h after drug administration, and the volume was replaced with sterile saline after each collection. Also, during the experiment, the animals had water ad libitum and were fasted by the time of the procedure up to 2 h after drug administration to avoid interference with absorption.

Plasma was obtained (centrifugation at 2500× *g* for 10 min) and stored at −70 °C for subsequent quantification of fexofenadine and pharmacokinetic evaluation. After completion of the pharmacokinetic sampling, animals were euthanized, and brains were subsequently collected for immunohistochemical analysis.

### 4.4. Pharmacokinetic Analysis of Fexofenadine

Fexofenadine was measured by ultra-high-performance liquid chromatography coupled with mass spectrometry (LCMS-8030, Shimadzu^®^, Tokyo, Japan), with electrospray ionization in positive mode (ESI+), with the following monitored mass-to-charge transitions: 502 → 484; 502 → 466; and 502 → 171. Losartan potassium (Sigma) was used as an internal standard and had the following mass-to-charge transitions monitored: 423 → 235; 423 → 207; 423 → 192.

The method was validated using a pool of white plasma (free of chemical substances) as a matrix, under the Food and Drug Administration (FDA) validation guideline [40] and presented a detection limit of 15.4 ng/mL of plasma, a quantification limit of 20 ng/mL of plasma, no matrix effect, no residual effect, and proved to be stable, precise, and accurate, with a linear range between 20 and 500 ng/mL.

The samples were prepared by protein precipitation with acetonitrile (LiChrosolv, Darmstadt, Germany) (1:10 plasma: acetonitrile), followed by ultracentrifugation at 22,000× *g* for 20 min. After centrifugation, 900 μL of the supernatant was collected and concentrated under vacuum at 40 °C until completely dry. It was then resuspended in 50 μL of mobile phase, and 25 μL were analyzed.

The mobile phase consisted of acetonitrile:methanol:ammonium acetate 10 mM (45:45:10 *v*/*v*/*v*), with a constant flow rate of 0.4 mL/minute. A C18 precolumn and C18 column (Shimadzu, Tokyo, Japan, model Shim-pack ODSPhenyl^®^, 3 mm × 100 mm × 2.2 µm) were used as the stationary phase. The oven was at 30 °C, with a total analysis time of 6 min. The gas flow of the nebulizer was 1.5 L/min, the DL temperature was 250 °C, the heating block temperature was 400 °C, and the drying gas flow was 15 L/min in the mass spectrometer.

The pharmacokinetic parameters (area under the curve (AUC), clearance (Cl), half-life (t_1/2_), maximum plasma concentration (C_max_), and time to reach maximum plasma concentration (t_max_)) were calculated based on the plasma concentration of fexofenadine over time using the trapezoidal method with WinNolin^®^ software (version 4.0 (PharsightCorp, Mountain View, CA, USA)).

### 4.5. Immunohistochemistry

After the last blood collection, the animals underwent the perfusion procedure to collect the brain; the sections were made in a microtome (Leica Biosystems, Wetzlar, Germany), with a thickness of 50 µm, at the region of interest, the hippocampus, which was subdivided into six sub-regions. For each brain, six coronal sections of each sample were made (2.12; 2.81; 3.50; 4.19; 4.88; 5.57 mm of Bregma) following the immunohistochemistry methodology adapted for the P-gp [28].

P-gp expression was assessed using a specific anti-P-glycoprotein antibody, and immunoreactivity was semi-quantitatively evaluated by densitometric analysis of staining intensity in hippocampal microvessels.

The hippocampus was selected as the region of interest due to its central role in limbic seizure propagation and epileptogenic remodeling, particularly in temporal lobe epilepsy. In the WAR model, animals exhibiting limbic recruitment (WAR-TLE phenotype) display seizure patterns involving forebrain structures, making the hippocampus a relevant anatomical substrate for investigating molecular alterations associated with chronic epilepsy. Furthermore, previous experimental and clinical studies on pharmacoresistance have consistently reported increased P-glycoprotein expression in hippocampal microvasculature. Therefore, the present analysis was designed to evaluate transporter expression in a region associated with epileptogenic progression rather than acute brainstem-mediated seizure initiation.

Although coronal sections encompassed both dorsal and ventral hippocampal regions, spatial distribution gradients of P-glycoprotein expression were not analyzed as independent variables. Instead, immunoreactivity was assessed using predefined hippocampal microvascular regions, and values obtained from different hippocampal levels were integrated to represent a single measurement per animal.

Regions of interest (ROIs) were defined over hippocampal microvascular structures for densitometric analysis. The green and blue ROIs shown in Figure 2 represent standardized sampling areas used to quantify P-gp immunoreactivity, ensuring consistency across sections and animals.

### 4.6. Statistical Analysis

For pharmacokinetic analyses, data were presented as median values. To compare the groups, the two-tailed Mann–Whitney test was used for unpaired and nonparametric data.

For immunohistochemical analyses, comparisons between groups were made using one-way analysis of variance (ANOVA) with Newman–Keuls post hoc testing.

Statistical tests were performed using GraphPadInStat^®^ software (version 3.10, San Diego, CA, USA), with a 95% confidence interval. The significance level for all statistical tests was set at 5%.

## 5. Conclusions

This study provides the first integrated molecular and functional characterization of P-glycoprotein in the WAR strain, a genetic model of epilepsy exhibiting phenotypic heterogeneity. By combining systemic pharmacokinetic analysis of a validated P-gp probe substrate with immunohistochemical assessment of transporter expression at the blood–brain barrier, our findings demonstrate that the WAR model is associated with both increased P-gp expression and altered drug disposition, particularly in animals with a refractory epilepsy phenotype.

Importantly, these results do not imply direct evidence of impaired antiepileptic drug efficacy but rather support the concept that transporter dysregulation represents a relevant molecular feature of this genetic epilepsy model. Therefore, the WAR strain emerges as a robust experimental platform for investigating regulatory mechanisms of P-gp, including transcriptional, epigenetic, and inflammatory pathways, as well as for testing pharmacological or molecular strategies aimed at modulating transporter activity.

Future studies integrating brain drug concentrations, pharmacodynamic outcomes, and selective transporter modulation will be essential to further elucidate the role of P-gp in epilepsy-associated pharmacoresistance and to translate these molecular insights into therapeutic strategies.

## Figures and Tables

**Table 1:** Pharmacokinetic parameters (median; range) of fexofenadine (10 mg/kg, PO, single dose) for control (Wistar), WAR-TLE, and WAR-RE animals.

Parameter	Control (Wistar)	WAR-TLE	WAR-RE
AUC (ng/mL·h)	577.9 (459.3–774.7)	779.5 (244.4–1314.6)	1200.8 (1031.1–1352) *
C_max_ (ng/mL)	151.6 (111.9–203.3)	298.5 (155.2–441.8)	296.5 (108.6–476.1)
t_max_ (h)	0.7 (0.4–0.8)	0.25 (0.25–0.25)	0.25 (0–1.9)
t_1/2_ (h)	4.2 (2.7–5.5)	6.4 (4.2–8.5)	11 (8.5–11.8) *
Cl_T/f_ (L/Kg/h)	17,396 (12,931–21,024)	17,985 (4856.5–31,113)	8330 (7288.9–9765.4) *

Legend: AUC: area under the curve; Cmax: maximum plasma concentration; tmax: time to reach maximum plasma concentration; t_1/2_: half-life; Cl_T/f_: apparent total clearance. * Mann–Whitney test, *p* < 0.05 compared with the control group.

## Data Availability

There are no additional data beyond those presented in this article. However, more detailed data can be requested from the authors.

## Abbreviations

The following abbreviations are used in this manuscript:

°C	Degrees Celsius
µm	Micrometer
ANOVA	Analysis of variance
AUC	Area under the curve
BBB	Blood–brain barrier
CEUA	Institutional Animal Care and Use Committee
ClT/f	Apparent total clearance
C_max_	Maximum plasma concentration
CNS	Central nervous system
dB	Decibel
ESI+	Electrospray ionization in positive mode
FDA	Food and Drug Administration
h	Hour
kg	Kilogram
L	Liter
mg	Milligram
miRNAs	MicroRNAs
mL	Milliliter
mm	Millimeter
ng	Nanogram
PET	Positron emission tomography
P-gp	P-glycoprotein
PO	Oral administration
t_1/2_	Half-life
t_max_	Time to reach maximum plasma concentration
UNIFAL-MG	Federal University of Alfenas
*v*/*v*/*v*	Volume/volume/volume
vs.	Versus
WAR	Wistar Audiogenic Rats
WAR-RE	WAR with refractory epilepsy
WAR-TLE	WAR with temporal lobe epilepsy
μL	Microliter
